# Experimental study of different dehydration methods in the process of preparing frozen brain sections

**DOI:** 10.1002/ibra.12075

**Published:** 2022-11-29

**Authors:** Hong‐Su Zhou, Jing Li, Yi‐Huan Guan, Hua He, Li‐Ren Huang Fu

**Affiliations:** ^1^ School of Anesthesiology Zunyi Medical University Zunyi Guizhou China; ^2^ Department of Experimental Animals Kunming Medical University Kunming Yunnan China; ^3^ Faculty of Health Sciences University of Adelaide Adelaide South Australia Australia

**Keywords:** cryosections, brain tissue, dehydration process, methodology, sucrose solutions

## Abstract

This study aimed to provide a recommendable protocol for the preparation of brain cryosections of rats to reduce and avoid ice crystals. We have designed five different dewatering solutions (Scheme 1: dehydrate with 15%, 20%, and 30% sucrose–phosphate‐buffered saline solution; Scheme 2: 20% sucrose and 30% sucrose; Scheme 3: 30% sucrose; Scheme 4: 10%, 20%, and 30% sucrose; and Scheme 5: the tissue was dehydrated with 15% and 30% sucrose polyacetate I until it sank to the bottom, followed by placement in 30% sucrose polyacetate II) to minimize the formation of ice crystals. Cryosections from different protocols were stained with Nissl staining and compared with each other by density between cells and the distance of intertissue spaces. The time required for the dehydration process from Scheme 1 to Scheme 5 was 24, 23, 24, 24, and 33 h, respectively. Density between cells gradually decreased from Scheme 1 to Scheme 5, and the distance of intertissue spaces was differentiated and irregular in different schemes according to the images of Nissl staining. We recommend the dewatering method of Scheme 4 (the brain tissues were dehydrated in 10%, 20% and 30% sucrose solution in turn until the tissue samples were completely immersed in the solution and then immersed in the next concentration solution for dehydration).

## Graphical abstract

Five Sprague–Dawley rats were anesthetized with isoflurane, and the brain tissue was removed for dehydration in the different sucrose concentrations and then prepared into brain slices. After Nissl staining, the images were observed and compared with each other.

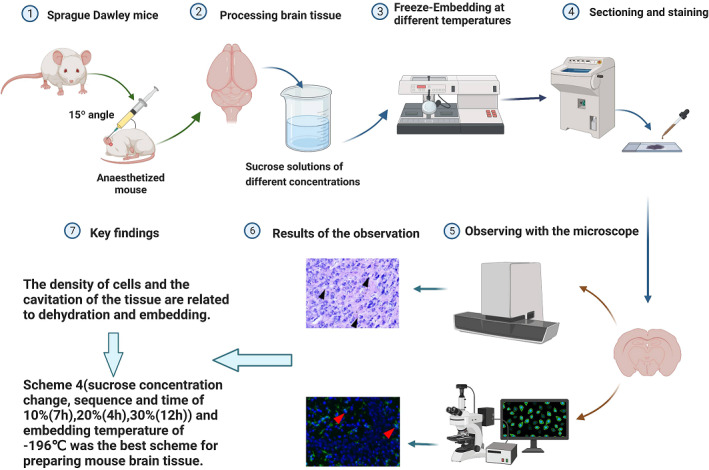

## 1 INTRODUCTION

Frozen section is a method of rapidly freezing tissue to a certain hardness for sectioning under low and constant cooling conditions. It plays an important role in both clinical tumor histopathology and animal experiments due to its advantages of rapidity, simplicity, and good preservation of antigenic activity and enzymes.^1^, ^2^, ^3^ High‐quality frozen sections are an important prerequisite to ensure the quality of the immunohistochemical process as well as image acquisition.^4^ The preparation process of frozen sections should involve different or the same factors of fixation, dehydration, and embedding according to the characteristics of different tissues (water content, texture, morphological structure, compositional diversity, and other factors). However, problems such as excessive tissue shrinkage or swelling, excessive brittleness, excessive ice crystal formation, and delamination are still unavoidable and have a significant impact on tissue cell morphology, which adversely affects the quality and results of frozen sections.^5^, ^6^, ^7^ The brain is loose, rich in water and phospholipids, and is prone to formation of ice crystals during the freezing process, which makes it difficult to detect brain tissue after ice crystal formation by deforming it under pressure, increasing the bias of experimental results.^8^, ^9^ Although a wide variety of methods are currently available for making frozen sections of brain tissue, and the overall level of production has shown great progress, there are still some detailed issues that deserve further study and discussion.^10^, ^11^, ^12^ The aim of this study was to prepare high‐quality frozen sections by observing the time required for each step after processing the brain tissue with different sucrose solutions and Nissl staining.

## 2 MATERIALS AND METHODS

### 2.1 Basic information

Five 7–8‐week‐old Sprague–Dawley rats (weighing approximately 270 g) were provided by Kunming Medical University and housed in a specific pathogen‐free (SPF) environment for Nissl and immunofluorescence staining (*n* = 5). All experiment rats were housed in plastic cages with a 12‐h light/dark cycle and free access to food and water following the guidelines of the US National Institutes of Health. The temperature of the rooms in which the animals were kept was maintained at 22  ±  1°C, with a relative humidity of 55  ±  5%. The study and all procedures were approved by the Animal Ethics Committee of Kunming Medical University (approval no. kmmu 2019011).

### 2.2 Instruments and equipment

**Table:** 

Name	Manufacturers	Lot. number (model)
37°C incubators	Shanghai Yuejin	DRP‐905Z
Pipette	Eppendorf (0.25) Dalong (10, 50, 200, and 1000 μ)	/
Inverted fluorescence microscopic camera system	Leica	2009030158
Cryostat	Leica	CM850
Digital slide scanner	Daniel	2020066771
Adhesion slide	Golden Bridge Biological Technology	/

### 2.3 Reagents

**Table:** 

Reagent name	Manufacturers	Batch no.	Storage method
Phosphate‐buffered saline (PBS)	Golden Bridge Biological Technology	ZLI‐9062	Ambient storage
Triton X‐100	Beyotime Biotechnology	0694	Ambient storage
4′,6‐Diamidino‐2‐phenylindole dihydrochloride (DAPI)	Beyotime Biotechnology	C1002	4°C
Antifluorescence quenching mounting medium	Beyotime Biotechnology	Po126	− 20°C
Nissl staining solution	Beyotime Biotechnology	150 ml	Ambient storage

### 2.4 Preparation of reagents

(1) 3% Bovine serum albumin (BSA) was prepared by measuring 97 µl of PBS with a pipette, adding 3 µl of BSA, mixing well, and storing at 4°C for later use.
(2) 0.2% Triton X‐100 was prepared by measuring 0.2 µl of Triton X‐100 with a pipette, adding 99.8 µl of PBS, mixing well, and storing at 4°C for later use.
(3) 1% Sheep serum was prepared by measuring 99 µl of PBS with a pipette, adding 1 µl of sheep serum, mixing well, and storing at 4°C for later use.
(4) 4′,6‐Diamidino‐2‐phenylindole dihydrochloride (DAPI) was prepared as DAPI:antifluorescent antiquench mounting medium = 1:3000.

### 2.5 Frozen section preparation

(1) After the rats were anesthetized with isoflurane, they were perfused systemically with 0.9% NaCl and 4% paraformaldehyde (PFA), and brain tissues were taken after the completion of perfusion.
(2) The brain tissues were fixed with 4% PFA for 24 h at 4°C.
(3) The fixed tissue was washed 1–2 times with PBS.
(4) Five different schemes (Table 1) for dehydrating, drying, and embedding optimal cutting temperature (OCT) compounds were designed. Animals were divided into five groups and subjected to each of the five schemes.
(5) The tissue block was fixed to the cryostat base.
(6) After trimming, the tissue was cut into cryosections with a thickness of 20 μm.
(7) The sections were attached to slides, infiltrated with PBS to prevent tissue folding, and the slides were baked overnight in a 60°C incubator.
(8) The slides were placed in a slicing box in sequence, marked with the tissue name, date, and so on, and stored in a −20°C refrigerator for preservation.

**Table 1:** Preparation of frozen sections for different schemes

Items	Scheme 1	Scheme 2^13^	Scheme 3^14^	Scheme 4^15^	Scheme 5^16^
The first dehydration	15% Sucrose–PBS solution (4 h)	20% Sucrose (11 h)	30% Sucrose (24 h)	10% Sucrose (7 h)	15% Sucrose polyacetate
The second dehydration	20% Sucrose–PBS solution (4 h)	30% Sucrose (12 h)	‐	20% Sucrose (4 h)	30% Sucrose polyacetate I
The third dehydration	30% Sucrose–PBS solution (8–10 h)	‐	‐	30% Sucrose (12 h)	30% Sucrose polyacetate II
Intervention	‐	‐	Cryoprotection	Cryoprotection	‐
Degree of Dehydration	Brain tissue sinks	Brain tissue sinks	Brain tissue sinks	Brain tissue sinks	Brain tissue sinks
Dry	After dehydration	After dehydration	After dehydration	After dehydration	After dehydration
OCT	−20°C	−80°C	−80°C	−196°C	−196°C

Abbreviations: OCT, optimal cutting temperature; PBS, phosphate‐buffered saline.

### 2.6 Nissl staining

(1) The brain slices were placed in a wet box, rinsed with PBS for 3 min × 3 times, and dropped into Nissl‐stained solution until the brain tissue was covered. The samples were mixed and stained with a pipette and stored at room temperature for 5 min, protected from light throughout the process.
(2) The staining solution was poured out and the brain slices were placed in a cylinder of distilled water and rinsed with running water for 1 min.
(3) The tissue was placed in 70% alcohol for differentiation.
(4) The tissue was rapidly dehydrated in absolute ethanol and then immersed in xylene to make the tissue clear and transparent, increasing the tissue refractive coefficient.
(5) After the brain slices were dried, the slices were sealed with neutral gum.
(6) Brain slices were examined using a fluorescence microscope and scanned using a digital sectioning scanner. It is important to note that stained specimens should be photographed as soon as possible and stained sections should be stored in the dark.

### 2.7 Immunofluorescence staining

(1) The tissue was removed and baked in a 37°C incubator for 30 min.
(2) The tissues were washed with PBS for 3 min × 3 times.
(3) The tissues were incubated for 2 h in PBS containing 0.2% Triton.
(4) The tissues were blocked with 3% BSA for 30 min at room temperature.
(5) Fifty microliters of a rabbit polyclonal antibody against Iba1 (Abcam; ab178846, 1:100) was dropped onto each brain tissue and incubated overnight at 4°C.
(6) The tissues were rinsed with PBS buffer for 3 min × 3 times. (The longer the culture time or the thicker the section, the longer the rinse time.)
(7) Sections were incubated with Alexa Fluor 488‐conjugated goat anti‐rabbit secondary antibodies for 2 h at room temperature.
(8) The tissues were rinsed with PBS buffer for 5 min × 3 times.
(9) Nuclei were counterstained with DAPI (DAPI:antifluorescence antiquenching mounting medium = 1:3000).
(10) Morphological changes were observed and photographed under a fluorescence inverted microscope.

### 2.8 Statistical analysis

**Figure 1:**
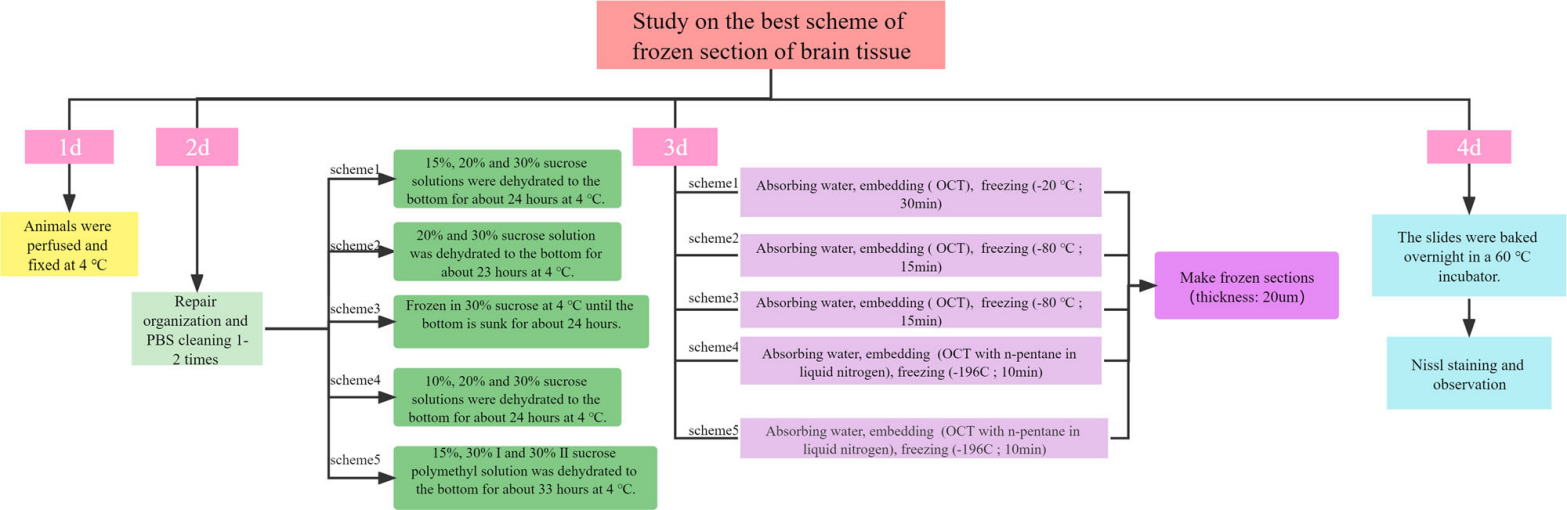
Procedure charts for different schemes [Color figure can be viewed at wileyonlinelibrary.com]

For this study, all hypotheses were tested using a two‐sided test, and the differences were considered statistically significant when *α* = 0.05 and *p* < 0.05. Quantitative variables were presented as means ± standard deviation. A two‐way analysis of variance (ANOVA), followed by the least significant difference test was used to compare differences in the number of ice crystals (NICs) from the sections of different schemes. All statistics were calculated using SPSS 22.0 (Figure 1).

## 3 RESULTS

**Figure 2:**
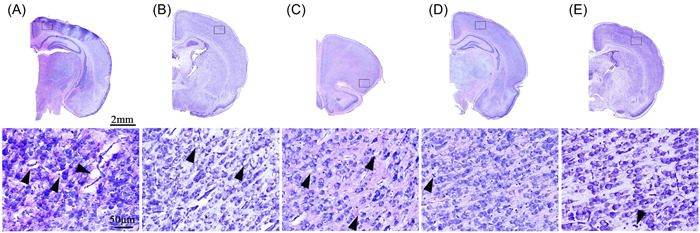
Nissl staining of frozen section with different schemes for rat brain. (A)–(E) show Nissl staining for frozen sections of Scheme 1, Scheme 2, Scheme 3, Scheme 4, and Scheme 5, respectively (ice crystals are shown by black triangles). [Color figure can be viewed at wileyonlinelibrary.com]

**Figure 3:**
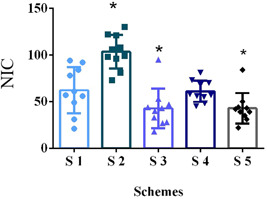
Number of ice crystals with the use of different schemes. S1, Scheme 1; S2, Scheme 2; S3, Scheme 3; S4, Scheme 4; S5, Scheme 5; NIC, number of ice crystals. *p < 0.05, versus Scheme 4. 5 samples in each scheme, two slices for each sample. [Color figure can be viewed at wileyonlinelibrary.com] [Correction added on 20 May 2024, after first online publication: The caption for Figure 3 was revised in this version at the request of the authors.]

**Figure 4:**
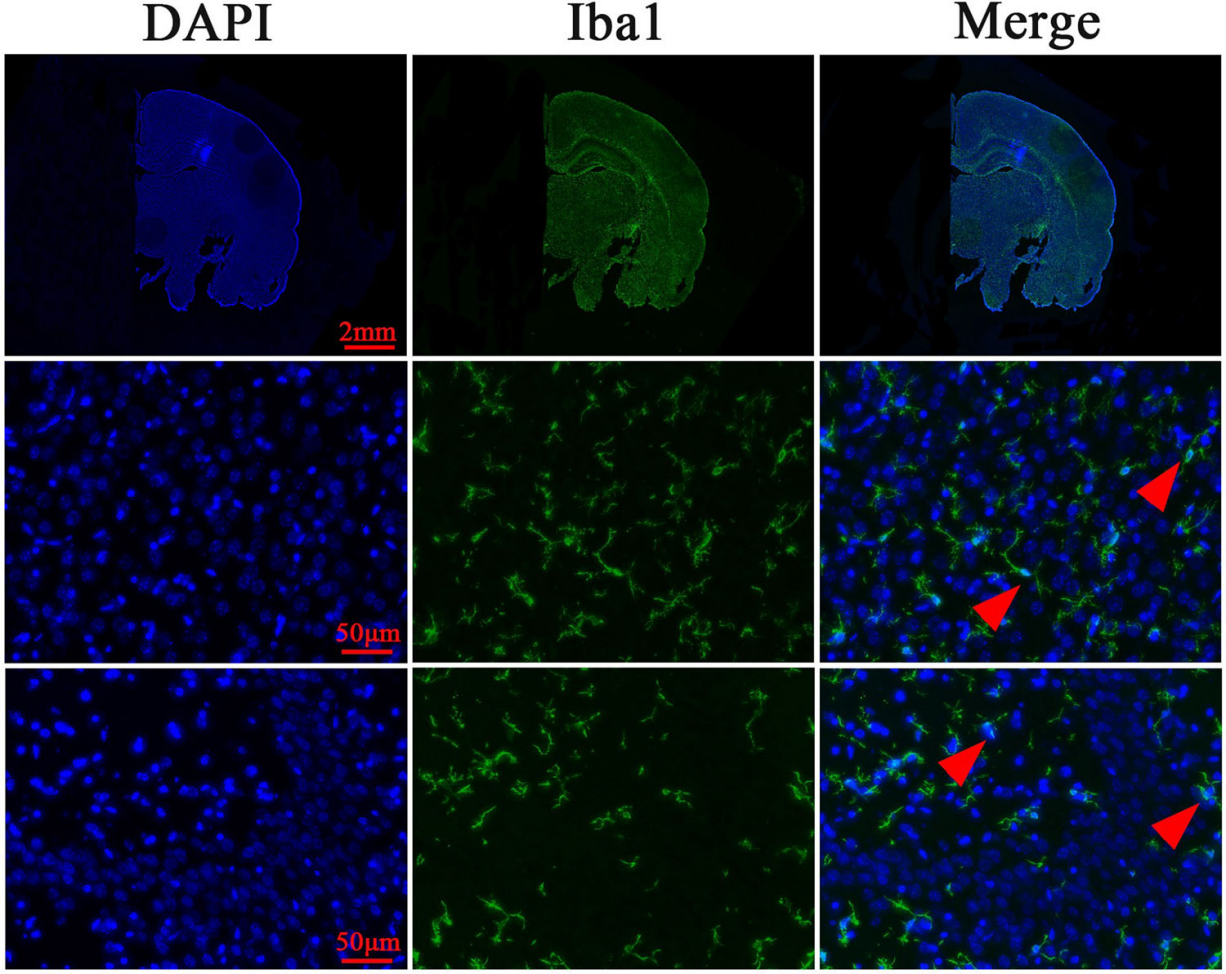
Immunofluorescence staining of frozen sections (Scheme 4). The red triangle shows the microglia. DAPI, 4′,6‐Diamidino‐2‐phenylindole dihydrochloride; Iba1, ionized calcium‐binding adapter molecule 1. [Color figure can be viewed at wileyonlinelibrary.com]

From each of the five groups, complete and unfolded frozen sections were selected. We observed that there was no excessive contraction and swelling of tissue sections. The slices were basically intact, without wrinkles, with a thickness of 20 μm, complete tissue structure, clear Nissl bodies, bright colors, and no obvious swelling or shrinkage of cells (Figure 2). The interneuron density gradually decreased, and the intercellular spaces were irregular; of these, the width of the intercellular spaces of Protocol 4 was the most suitable. In addition, Scheme 1 had the longest embedding time at 30 min, Scheme 5 took up to 33 h in the dehydration stage, and there was no significant difference between the other schemes (Table 2). NIC was significantly reduced in Scheme 1 (62.30 **±** 24.80), Scheme 3 (42.1 **±** 21.17), Scheme 4 (61.10 **±** 11.47), and Scheme 5 (42.9 **±** 16.37) compared to Scheme 2 (103.40 **±** 18.01) (*p* **<** 0.01, Figure 3). NIC was not different between Scheme 1 and Scheme 4 (*p* **=** 0.888). Immunofluorescence staining of the frozen section of Scheme 4 showed microglia with a clear background, elongated and branched cell body, and many small spinous processes on the surface (Figure 4).

**Table 2:** Results were obtained with the use of different schemes

Scheme	Dehydration time (h)	Embedding time (min)	Nissl staining effect	
			Cell density	The void among tissue
Scheme 1	24	30	+ + + + +	+ + +
Scheme 2	23	15	+ + + +	+ +
Scheme 3	24	15	+ + + +	+ + +
Scheme 4	24	10	+ + +	+
Scheme 5	33	10	+ +	+ +

*Note*: “+” represents the density of cells and tissue voids.

## 4 DISCUSSION

In this study, the whole‐brain tissue of rats was sampled. After 24 h of fixation, the frozen sections of mice brain tissues were prepared using the method of our laboratory (Scheme 1) and other laboratory methods were used. Scheme 4 avoided tissue contraction, tissue hardening and increased brittleness, which due to prolonged dehydration while ensuring adequate dehydration and transparency of the tissue, although the amount of NICs produced was not the least among all schemes. Moreover, the results of Nissl and immunofluorescence staining showed uniform and clear Nissl body morphology, and significant microglia, elongated and branched processes, and small spinous processes.

It is well known that recovery from diseases related to the nervous system takes a lot of time. It may even remain irreversible. For a long time, improvement of neural recovery has been one of the focuses and hot spots in the research field. More importantly, scientists and researchers have also made a great deal of merit and achievements in the nervous system, bringing a light to many complex and confusing diseases.^17^, ^18^, ^19^ The establishment of animal models of different diseases plays an irreplaceable role in the process of gradually transferring scientific research achievements from laboratory to ward.^20^, ^21^ Imperceptible changes in each experimental technique may be the key to success. Therefore, we should observe the effects of changes in various experimental conditions on the experimental results meticulously and scientifically, even during the preparation of tissue slices. For the nervous system, brain cytoarchitecture is a complex assemblage of multiple cell types, supported by an intricate microvascular network. Neurons, astrocytes, microglia, oligodendrocytes, endothelial cells, and so on have a molecular signature and morphology that defines their type and functional state (e.g., resting, reactive, proliferating, apoptotic, phagocytic, etc.).^22^ In system brain histology studies, simultaneous profiling of multiple biological processes is carried out in their native anatomical context, which has greatly enabled neuroscience research and development. In the methodology for the frozen section preparation described in this paper, we further confirmed that the sucrose concentration and embedding temperature of Scheme 4 (tissue dehydration was performed in 10%, 20%, and 30% sucrose solutions to remove water from the tissue, followed by cryoprotection and embedding at −196°C) were most favorable for Nissl staining of brain tissue. In fact, many laboratories often use the same dehydration scheme to conduct experiments on different organs and tissues, resulting in the inability to update the scheme in time to understand the scheme that is more conducive to the progress of the experiment, which is also not rigorous. On the one hand, for different tissues and organs, customized dehydration measures should be performed according to their specific characteristics.^23^, ^24^ If the dehydration time is too long, the interstitial space increases, while if the dehydration time is too short, water in the tissue cannot be effectively replaced by paraffin and sections cannot be prepared. In other words, a customized dehydration program can help to better maintain the shape and avoid the problems of fragile tissue, incomplete wax slices, and uneven thickness during embedding caused by incomplete dehydration. On the other hand, even the same tissue may be processed differently to some extent depending on the subsequent experiments (such as paraffin sections and frozen sections), so generalizations cannot be made.^24^ In addition, from the results of this experiment, we found that with the use of the gradient sucrose solution, the morphology of the tissue could be better maintained. The sucrose concentrations that we used previously were 15%, 20%, and 30%. Rahman et al.^14^ utilized only a 30% sucrose solution, while Maric et al.^15^ also tried combinations of 15%, 20%, and 30% sucrose concentrations. In 2021, Maric et al. published a paper in *Nature* describing the major types of cells observed in the same whole‐brain section, and eventually, using immunofluorescence, 3 dimensional imaging of the neural network is demonstrated.^22^ This undoubtedly required higher‐quality frozen sections, which were indeed processed using Scheme 4 mentioned in our paper. The time spent in dehydrated tissue varies with the concentration of sucrose solution, and it is difficult to guarantee that a solution of a particular concentration gradient will necessarily be suitable for a particular tissue, but it is possible to screen out the better from the different options. In sum, to improve the quality of slide preparation and improve efficiency, this study focused on comparison of different dehydration methods of rat brain, performed by varying the sucrose concentration and adjusting the corresponding time. At the same time, after verification and comparison with the methods of other laboratories, it was proven that the five schemes mentioned in this paper were applicable. Finally, through the evaluation of the effect of Nissl staining, the dehydration method with better practicability and guiding significance was summarized. It not only homogenizes the density between cells for observation but also prevents the formation of ice crystals in frozen sections considerably.

The formation of ice crystals in frozen sections is a problem that should be avoided and it is a major factor that affects accurate diagnosis because ice crystals lead to the formation of blank areas in the slices and the number of intracellular vacuoles increase, making it difficult to distinguish the tissue structure and also making diagnosis difficult.^25^ In particular, tissues with liquid components tend to form ice crystals, such as brain tissue, which is also one of the most troublesome tissues to freeze sections. We usually prefer to use OCT embedding agents without moisture. In addition, temperature control is a key to successful preparation of frozen sections. We have tried many measures and achieved improvements in this regard, for example, each time the sampling board is wiped dry with gauze as much as possible before embedding dry tissue moisture and quick freezing.

Liquid nitrogen quenching is the best method for rapid tissue freezing, but uneven freezing can lead to brain tissue rupture. The common method is to wrap an appropriate amount of OCT around the brain tissue and then directly pour liquid nitrogen on the sample or place the sample with the sample holder in liquid nitrogen.^16^, ^26^ In addition, it is necessary to slow down and then fast when sectioning. Because of this, on the one hand, slow cutting of the brain at the beginning can reduce the cutting force of the blade, and the slice is not easy to curl. On the other hand, with fast brain sectioning, the section can be quickly removed from the blade and tightly fitted to the glass slide. Furthermore, the slides can be infiltrated with PBS during the section to prevent tissue wrinkles. Therefore, after sampling, the tissue should be gently dabbed with dry gauze to remove the water as much as possible, which can reduce the formation of excessive ice crystals after freezing due to excessive water in the tissue to a certain extent. When embedding, the thickness of the embedding agent drops on the freezing tray can be increased accordingly compared with the general tissue, so that the tissue is not too close to the freezing tray, which can prevent excessive freezing and effectively reduce the formation of ice crystals at the same time. Meanwhile, the freezing temperature should be set at −20°C and the freezing time should also be strictly controlled by monitoring the temperature regularly. To avoid freezing, we can also remove the freezing table when it is about to freeze. When performing slicing, we cut off the unfrozen tissue and discarded it. It is advisable to select the tissue that has just been frozen. In sum, unsuitable temperatures during slicing, which will lead to insufficient tissue hardness, and the sections will appear stacked and uneven thickness. At the same time, the friction force during slicing will be increased, making it difficult to perform slicing; during immunofluorescence staining, maintaining room temperature when blocking and incubating the secondary antibody can help reduce background staining. In short, the above steps can help prevent the formation of excessive ice crystals in frozen sections.

## 5 CONCLUSION

Taken together, the density of cells and their interstices correlate with the dehydration and embedding processes. After intense discussion among experienced experimental technicians and scientific researchers, it was agreed that among the above schemes, Scheme 4 provided a dehydration procedure around sucrose solution, and the effect was very ideal, which was worth trying.

## AUTHOR CONTRIBUTIONS

Hua He and Li‐Ren Huang Fu recorded the experimental data and conducted the experiments. Hong‐Su Zhou, Yi‐Huan Guan, and Jing Li wrote the manuscript. Li‐Ren Huang Fu polished the whole article. All authors have read and approved the final submitted manuscript.

## CONFLICT OF INTEREST

The authors declare no conflict of interest.

## ETHICS STATEMENT

All experimental protocols were approved by the Animal Care and Use Committee of Kunming Medical University in Yunnan Province, China. The application number for animal experiments ethical inspection: No. kmmu2019011. Animal handling procedures conformed to the National Institutes of Health Guide for the Care and Use of Laboratory Animals.

## Data Availability

The data sets used and/or analyzed during the current study may be obtained from the corresponding authors upon reasonable request.
